# Phosphorus mining and bioavailability for plant acquisition: environmental sustainability perspectives

**DOI:** 10.1007/s10661-025-14012-7

**Published:** 2025-04-21

**Authors:** Matthew Chidozie Ogwu, Micaela Elizabeth Patterson, Pia Angelina Senchak

**Affiliations:** 1 Goodnight Family Department of Sustainable Development, Living Learning Center, Appalachian State University, 212, 305 Bodenheimer Drive, Boone, NC 28608 USA; 2 Department of Geological and Environmental Sciences, Appalachian State University, Rankin Science West, Boone, NC 28608 - 2067 USA

**Keywords:** Phosphorus sustainability, Heavy metal contamination, Phosphorus fixation, Rhizosphere interactions, Organic waste recycling

## Abstract

This review aims to examine microbial mechanisms for phosphorus (P) solubilization, assess the impacts of P mining and scarcity, and advocate for sustainable recycling strategies to enhance agricultural and environmental resilience. Phosphorus is an indispensable macronutrient for plant growth and agricultural productivity, yet its bioavailability in cultivation systems is often constrained. This scarcity has led to a heavy reliance on fertilizers derived from mined phosphate rock (PR), which is a finite resource usually contaminated with hazardous elements such as uranium, radium, and thorium. Plants absorb only about 10–20% of P from applied fertilizers, leading to significant inefficiencies and negative environmental consequences. Additionally, the uneven geographic distribution of PR reserves exacerbates global socioeconomic and geopolitical vulnerabilities. Healthy soils enriched with diverse microbial communities provide a sustainable avenue to address these growing challenges. Rhizospheric organisms, including phosphorus-solubilizing and phosphorus-mineralizing bacteria and arbuscular mycorrhizal fungi, are capable and pivotal in the sustainable conversion of inorganic and organic P into bioavailable forms, reducing reliance on synthetic fertilizers. The mechanisms used by these microbes often include releasing organic acids to lower soil pH and solubilize insoluble inorganic phosphorus compounds and the production of enzymes, such as phosphatases and phytases, to break down organic phosphorus compounds, including phytates, into bioavailable inorganic phosphate. Some microbes secrete chelating agents, such as siderophores, to bind metal ions and free phosphorus from insoluble complexes and use biofilms for P exchange. This review also advocates for the recycling second-generation P from organic waste as a sustainable and socially equitable alternative to conventional phosphate mining.

## Introduction

Phosphorus (P), a finite and irreplaceable resource, is the backbone of global food production, yet its unsustainable use has placed humanity on the brink of a “phosphorus crisis.” With over 85% of the world’s P reserves concentrated in a handful of countries, the growing demand for phosphate fertilizers threatens food security and geopolitical stability. Furthermore, inefficient P utilization has led to environmental degradation, including eutrophication of water bodies, making its sustainable management a pressing global challenge (Holtan et al., 1988). It plays a key role in cellular processes such as energy transfer, signal transduction, and nucleic acid and phospholipid synthesis (Castagno et al., 2021). While P is abundant in the Earth’s crust, it often exists in insoluble inorganic and organic complexes, making it inaccessible primarily to plants (Barrow, 2022). The bioavailability of P is a critical factor influencing plant growth and agricultural productivity.

In soils, P is primarily present in organic forms and is associated with soil minerals. The availability of P to plants is influenced by various chemical, biological, and physical processes that release P from these complexes (Jungk, 2002; Tinker & Nye, 2000). Ultimately, plants rely heavily on rhizospheric microbiota to access P, incredibly complex communities containing phosphate-solubilizing bacteria (PSB), phosphate-mineralizing bacteria (PMB), and arbuscular mycorrhizal fungi (AMF), which facilitate phosphorus solubilization and uptake through the secretion of enzymes and organic acids (Barrow & Lambers, 2022; Sharma et al., 2013). These microorganisms are pivotal in transforming insoluble P compounds into bioavailable forms, enhancing plant nutrient acquisition (Gerke, 2015; Liu et al., 2022a, b). These microorganisms secrete organic acids and enzymes that convert insoluble P into forms that plants can absorb. Recent studies have highlighted the importance of the interaction between plants and rhizospheric microbiota in improving soil fertility, P acquisition, and microbial diversity in maintaining plant health (Blackwell et al., 2019; Dolatabadian, 2020; Konečný et al., 2019; Pantigoso et al., 2022). For instance, Konečný et al. (2019) investigated the role of specific genes involved in the exchange of carbon and P between plants and AMF, demonstrating that AM symbiosis can significantly enhance P uptake by plants. The study underscores the potential of microbial inoculants and genetic strategies to improve P-use efficiency in crops.

**Fig. 1:**
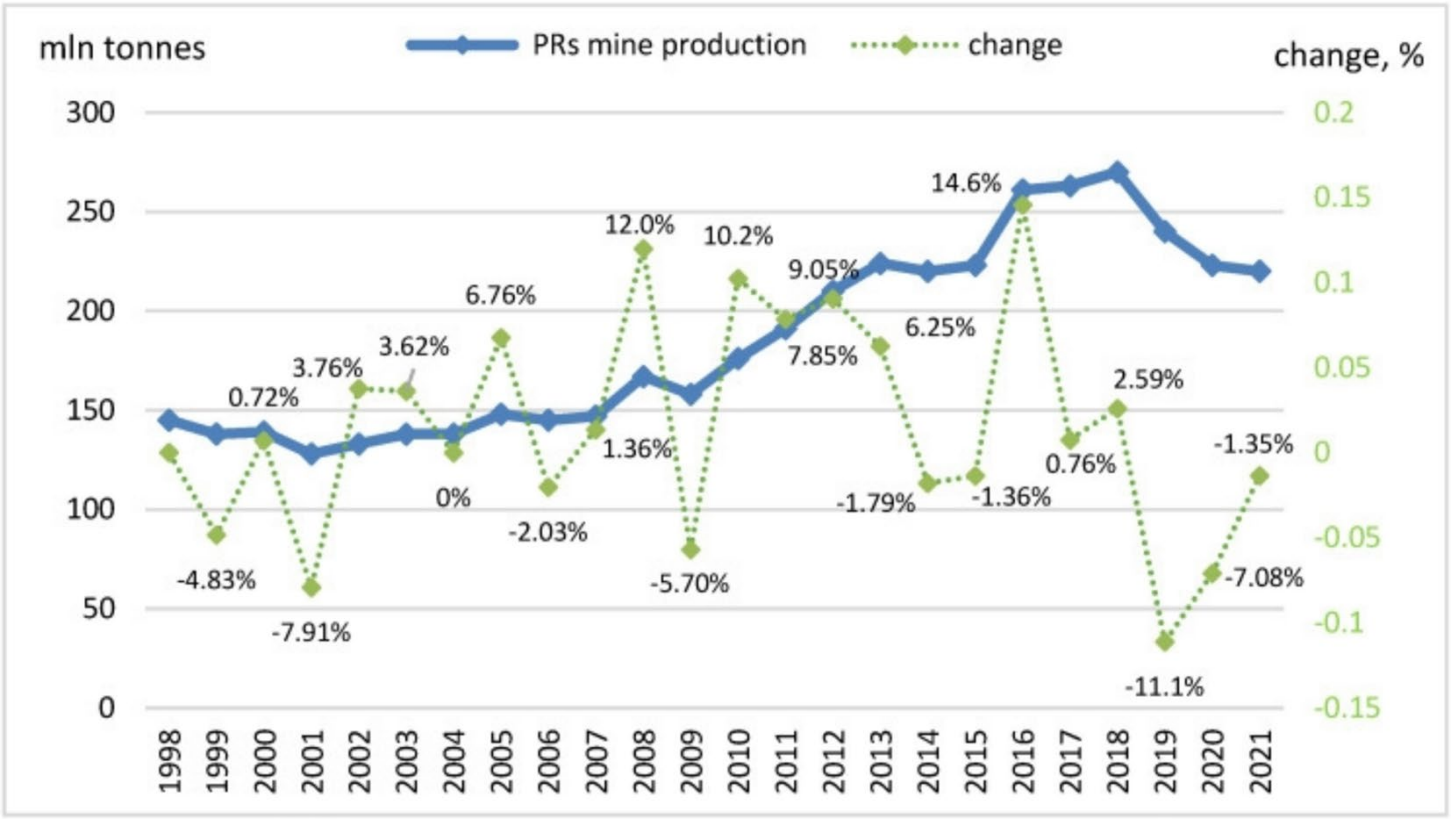
Global trend in phosphate rock mining operations between 1998 and 2021. Source: U.S. Geological Survey (2022)

While biological mechanisms offer a sustainable pathway for P acquisition, modern agriculture predominantly depends on P fertilizers derived from mined phosphate rock (PR), mostly from countries listed in Table 1. Phosphate rock, predominantly found as sedimentary or igneous deposits, is the primary source of P fertilizers (Ryszko et al., 2023). The geopolitical distribution of these PR reserves, concentrated in Morocco, China, and Russia, further complicates global P supply chains (Schoumans et al., 2015). Production values have historically fluctuated but continue to experience steady growth (Fig. 1). Fluctuations in PR prices and supply disruptions, exacerbated by geopolitical tensions, highlight the urgent need for sustainable P management strategies (Scholz & Wellmer, 2021). With concerns over the finite nature of PR reserves, exploring alternative sources and recycling methods is imperative to ensure long-term P sustainability (Nedelciu et al., 2020; Zwetsloot et al., 2015a, b). The extraction and processing of PR contributes to the depletion of finite P reserves and introduces heavy metals and radioactive elements into the environment, posing significant ecological and health risks (Mew et al., 2018).

**Table 1:** Estimates of world phosphate rock production in 2021

Country	Million tons
USA	25
Morocco	150
China	110
Russia	155
Other countries	220

Source: Adapted from Ryszko et al. (2023)

Innovative approaches to P recycling are gaining attention as viable solutions to mitigate the environmental impacts of P mining. Technologies such as struvite precipitation, hydrothermal treatment, and microbial inoculation offer promising avenues for recovering P from waste streams (Jastrzębska et al., 2022; Witek-Krowiak et al., 2022). Recycling P from agricultural and urban wastes reduces reliance on mined resources and addresses issues of eutrophication and nutrient runoff in aquatic ecosystems (Ryden et al*.,* 1977; Schaedig et al., 2023).

These processes promote two essential concepts—"first-generation mined P,"referring to P extracted directly from PR, which has limited sustainability due to its environmental and geopolitical challenges, and"second-generation P,"which involves the recycling of P from organic waste materials, offering a more sustainable alternative (Nedelciu et al., 2020). Phosphorus recycling from agricultural and urban wastes can reduce the dependence on mined P and mitigate its negative environmental impacts (Jayathilakan et al., 2012; Metson et al., 2022). Furthermore, excessive use of P fertilizers contributes to environmental issues such as eutrophication, where runoff leads to nutrient overload in aquatic systems, causing harmful algal blooms and dead zones (Schaedig et al., 2023). Addressing these challenges requires a shift toward more sustainable P management practices, including the use of microbial solutions and the recovery of P from waste streams (Wang et al., 2023).

This review assesses the environmental impacts of P mining and explores sustainable strategies for improving P bioavailability and recycling. By highlighting the role of rhizospheric microorganisms and the potential of P recovery technologies, this review seeks to promote resilient and sustainable agricultural systems that reduce reliance on the likely finite PR resources.

## Economic and social implications of phosphorus scarcity

Phosphorus scarcity has far-reaching economic and social implications, particularly in agriculture, directly impacting productivity and food security. The rising costs of P-based fertilizers pose significant challenges for farmers, especially in resource-limited regions where profit margins are already precarious. Surging phosphate rock prices have increased operational costs, forcing many farmers to adopt unsustainable practices or scale back operations, further threatening their livelihoods and long-term agricultural productivity (Brownlie et al., 2023; Cordell & White, 2015). These pressures are particularly acute for smallholder farmers in developing countries, where limited financial resources compound the effects of rising input costs, creating a vicious cycle of poverty and food insecurity (Cordell & White, 2013, 2015).

From a geopolitical perspective, the global supply of phosphate rock is concentrated in a handful of countries, including Morocco, China, and Russia. This uneven distribution creates significant vulnerabilities in the global supply chain, leaving countries without local P sources reliant on a small number of suppliers. Such dependency increases the risk of supply disruptions and geopolitical tensions, especially as global demand for P is projected to outpace supply in the coming decades (Jasinski, 2018; Nedelciu et al., 2020). Moreover, while P recycling technologies present a promising avenue for reducing dependence on mined phosphate rock, the financial burden of adopting these innovations remains a barrier. The initial investment in such technologies is prohibitively expensive for many farmers and industries, particularly small-scale operations, despite their long-term sustainability benefits (Cordell & White, 2013; Neset et al., 2016). The social implications of P scarcity are equally concerning because of P insecurity, i.e., the vulnerability of agricultural and food systems to the challenges associated with the availability, accessibility, and sustainable use of P. Rising fertilizer costs exacerbate inequalities in agricultural productivity, disproportionately affecting smallholder farmers who lack access to affordable inputs. This inequity widens the gap between wealthier farmers who can maintain or improve yields and poorer farmers who struggle to remain viable (Cordell & White, 2013, 2015). The downstream effects include heightened food insecurity, particularly in regions already grappling with malnutrition and limited access to nutritious food. Declining agricultural productivity driven by P scarcity can devastate global health, increasing the prevalence of undernourishment and associated health conditions (Cordell & White, 2015).

Addressing these challenges requires coordinated global efforts to promote equitable P management and sustainable alternatives. Policies that support smallholder farmers, such as subsidies for biofertilizers or low-cost P recovery methods, can help mitigate financial barriers and foster resilience in vulnerable communities (Neset et al., 2016). Additionally, international collaboration is crucial to ensure equitable access to P resources and to drive investments in technologies that enhance P recycling and efficient use. Public awareness campaigns emphasizing responsible P use and recycling at the community level can also play a pivotal role in fostering a culture of sustainability and conservation (Cordell & White, 2013; Neset et al., 2016).

## Environmental and human health implications of phosphorus mining

Phosphate rock is predominantly mined using surface methods, including draglines and bucket wheel excavators for large deposits and power shovels or earthmovers for smaller deposits (Mew et al., 2018; Mwalongo et al., 2023a). In some cases, underground mining methods, such as the room-and-pillar technique, are employed, similar to coal mining. Most PR mining occurs in the Western Sahara region of Morocco, which significantly influences the global P supply chain. This region holds the largest reserves, estimated at 50,000 million metric tons, accounting for approximately 70% of the world’s supply. Other significant contributors are China (3200 million metric tons), the USA (1000 million metric tons), and Jordan (1000 million metric tons). Saudi Arabia, South Africa, Egypt, and Australia also have substantial reserves ranging between 1000 and 2800 million metric tons, with companies like Ma’aden and Foskor leading production. Countries like India, with limited domestic reserves (46 million metric tons), rely heavily on imports to meet demands. These concentrated reserves and infrastructures highlight the geopolitical and economic significance of phosphate mining and its critical role in agriculture and industry. The environmental consequences of phosphate mining are substantial, with biogeosociochemical impacts including toxic mineral runoff, soil erosion, acid mine drainage, and contamination from tailing impoundments and heap leaching. Phosphate rocks are often associated with toxic and radioactive minerals such as arsenic, chromium, lead, uranium, mercury, nickel, and cadmium (Reta et al., 2018).

**Fig. 2:**
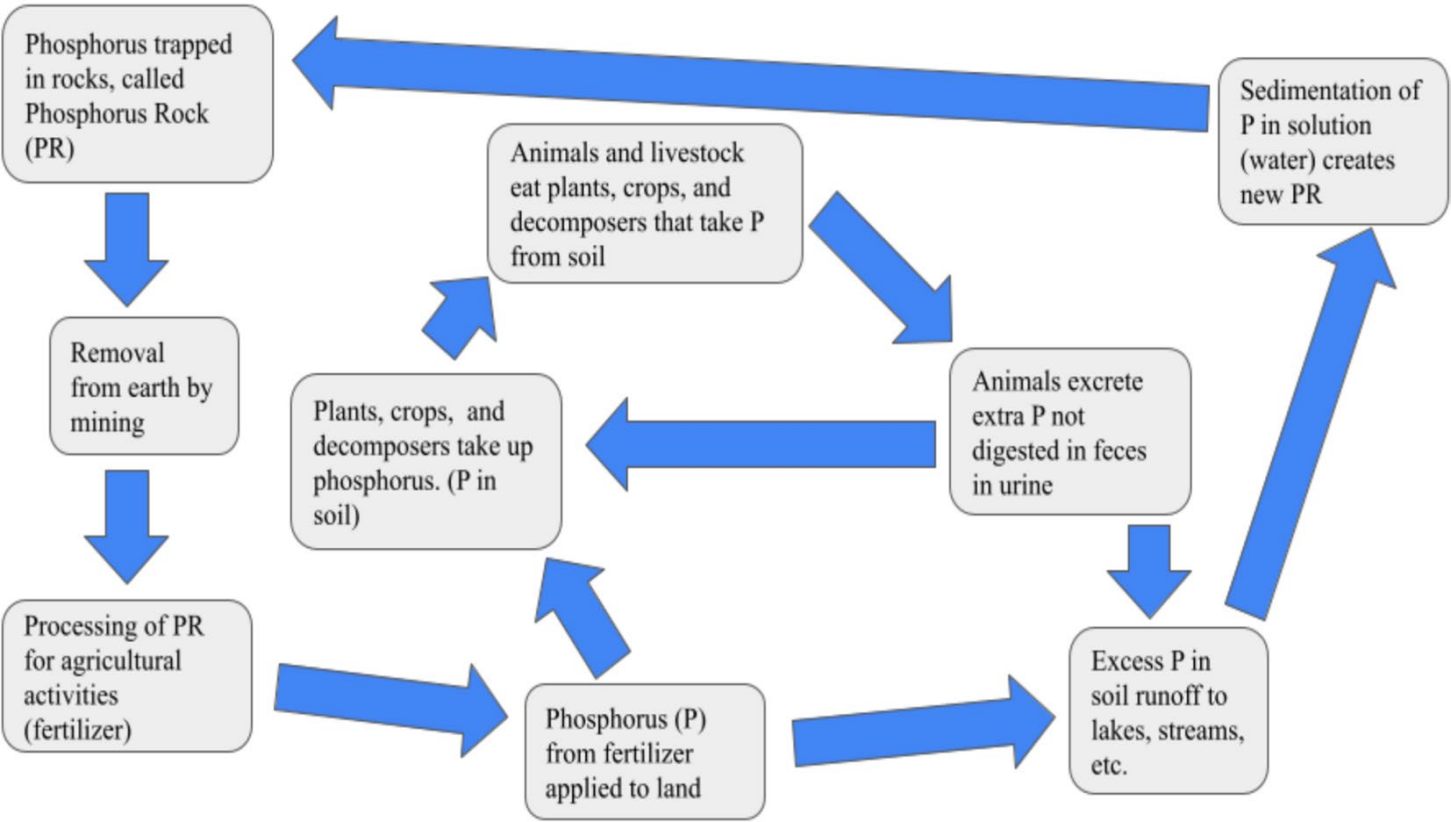
Simplified model of the global P flow diagram

PR processing for fertilizer production primarily involves wet phosphoric acid processing, which generates phosphogypsum as a byproduct (Ryszko et al., 2023). This phosphogypsum is radioactive and requires careful storage to prevent environmental contamination. Improper handling and storage of phosphogypsum can lead to environmental disasters, as exemplified by the Piney Point phosphogypsum spill in Tampa Bay (Florida, USA), which contributed to red tide events (Beck et al., 2023; Rhodes, 2013). The global P cycle comprises several key components (Fig. 2):

1. Tectonic uplift and exposure of P-bearing rocks to weathering forces.
2. Physical erosion and chemical weathering of rocks, resulting in soil formation and the provision of dissolved and particulate P to rivers.
3. Riverine transport of P to lakes and oceans.
4. Sedimentation and burial of P with organic and mineral matter in sediments.

Human activities, particularly phosphate mining, disrupt the natural P cycle by altering the amounts of P entering terrestrial and aquatic ecosystems. The extraction and processing of PR result in P losses from marine biospheres, as sediments on the ocean floor accumulate P as insoluble calcium phosphate. Although geological processes can remobilize some P, there remains a net annual loss of millions of tons from marine ecosystems (Hao et al., 2017; Mghazli et al., 2021).

Phosphate rock is a sedimentary rock containing 4–20% phosphorus pentoxide (P_2_O_5_), the primary ingredient in P fertilizers. Many US phosphate deposits, particularly those in the Roseland District of Virginia, were formed during the Neogene period. While phosphate rocks are not commonly found in the Appalachian Mountains (USA), they are more prevalent in the central to coastal regions of the southeastern United States, mainly from North Carolina to Florida (Swaby et al., 2016). Although PR mining is not active in the Appalachian region, it plays a crucial role in the area’s agricultural economy.

The mining of PR disturbs natural P cycles by altering P distribution in natural sinks and contributing to eutrophication through runoff (Barrow, 2022; Barrow & Lambers, 2022). This mining is primarily done through.

1. Surface (open pit) methods using draglines and bucket wheel excavators for large deposits and power shovels or earthmovers for smaller deposits.
2. Underground mining that employs room-and-pillar methods like coal mining.

Mining of phosphate rock often introduces various trace elements and contaminants into the environment, some of which pose significant risks to human health and ecosystems. These contaminants either naturally occur within the phosphate deposits or result from mining and processing activities. Table 2 provides an overview of the average concentrations of key constituents found in PR, highlighting the potential environmental and health challenges associated with P extraction and utilization. This information underscores the importance of adopting sustainable practices to mitigate contamination risks. Treating mined PR with sulfuric or oxalic acid to produce phosphoric acid generates phosphogypsum, a byproduct associated with red tides. Significant radioactivity levels have been documented in crops such as maize and mung beans due to contaminant exposure from phosphate fertilizers (Mwalongo et al., 2023b, 2024). Removing contaminants like uranium from PR is not economically feasible, as many countries lack regulations on uranium concentrations in fertilizers (Yu et al., 2020). In the USA, critical soil contamination levels for uranium are set at 30 mg/kg, while Canada has set this limit at 23 mg/kg (Alewel et al. 2020). The updated European Union fertilizer legislation (Regulation EU 2019/1009) bans the sale of inorganic fertilizers with specific toxic contaminants, such as lead, mercury, hexavalent chromium, nickel, arsenic, and cadmium, in amounts exceeding set maximum concentration values. However, uranium content remains unregulated (Alewel et al., 2020).

**Table 2:** Contaminants in mined phosphorus and their average concentrations

Constituent	Average concentration in phosphate rock (mg/kg)
Arsenic	13.37
Cadmium	15.42
Chlorine	463.00
Total chromium	156.3
Hexavalent chromium	0.47
Copper	22.70
Mercury	0.03
Manganese	36.28
Nickel	28.20
Lead	< 8.00
Titanium	85.20
Uranium	226.48
Vanadium	107.24
Zinc	270.60

Source: Adapted from Mwalongo et al. (2023a)

The mineral composition of PR, detailed in Table 3, is critical for determining its quality and economic viability for fertilizer production. This composition also influences the presence of contaminants in fertilizers derived from these rocks.

**Table 3:** Major element concentration in phosphate rock

Constituent	Average concentration in phosphate rock (wt.%)
Diphosphorus pentoxide	29.74
Calcium oxide	53.58
Aluminum oxide	0.38
Fluorine	3.50
Iron (III) oxide	0.80
Potassium oxide	0.17
Magnesium oxide	0.68
Sodium oxide	0.87
Sulfur trioxide	2.51
Silicon dioxide	4.34
Strontium oxide	0.22

Source: Adapted from Mwalongo et al. (2023a)

It is important to note that precise fertilizer formulations are essential for optimal yields for industries such as Christmas tree farming in the Appalachian High Country. For agriculture, cereals like wheat benefit from NPK [i.e., nitrogen:phosphorus:potassium] (20–10 - 10), which promotes leaf growth, root development, and grain production (Godebo et al., 2021; Makhdum et al., 2024). In horticulture, fruits such as citrus thrive with NPK (15–5–30), enhancing fruit size, quality, and overall plant health (Ma et al., 2022). Forestry applications like pine timber require slow-release fertilizers (18–6–12) to support sustained growth and strong root systems (Barłóg et al., 2022). Landscaping uses turf fertilizers (25–5–10) for lush, resilient turfgrass. Aquaculture relies on balanced NPK (10–10 - 10) to promote aquatic plant growth and ecosystem balance (Huang et al., 2020). Greenhouse crops like tomatoes and peppers perform best with soluble NPK (20–20 - 20) for balanced nutrition, while ornamental flowers benefit from high-P fertilizers (10–30 - 20) to boost flowering and bloom quality. Each formulation is tailored to specific crop requirements, ensuring optimal growth and productivity. For P fertilization, diammonium phosphate (18–46 - 0 N-P-K ratio) and concentrated superphosphate (0–46 - 0 N-P-K ratio) are recommended, particularly when incorporated into the soil early in crop rotations to build P levels (NC State Extension, 2023). Phosphorus recommendations based on soil testing are provided in Table 4.

**Table 4:** Sample soil P_2_O_5_ and K_2_O testing spectrum and application suggestion

Spectrum soil testing	P_2_O_5_ lbs/acre to apply	K_2_O lbs/acre to apply
Low	120	120
Medium	80	80
Good	40	40
High	0	0

*P*_*2*_*O*_*5*_; diphosphorus pentoxide, *K*_*2*_*O;* dipotassium oxide, *lbs;* pounds

Phosphorus is a critical nutrient for human health, essential for bones, teeth, deoxyribonucleic acid, and ribonucleic acid. However, excess P intake can lead to health issues, such as hypocalcemia, characterized by weak bones due to calcium being pulled from them (Matej-Lukowicz et al., 2020; Trautvetter et al., 2018). Examples of high-P foods include whole-grain bread, bran cereals, nuts, and dark-colored sodas.

## Sustainable phosphorus recycling techniques

The conventional method of sourcing P by mining PR is unsustainable due to the finite nature of these reserves and the environmental impacts associated with mining activities. Consequently, there has been increasing interest in sustainable P recycling techniques that can recover P from waste streams, reduce reliance on PR, and mitigate environmental pollution. Second-generation P recovery refers to environmentally sustainable methods of obtaining P from waste materials, as opposed to traditional mining of PR, which is considered first-generation P sourcing (Hollas et al., 2021a, b). These recovery methods include biological, chemical, technological, and electrochemical processes applied to solid, semi-solid, or liquid waste byproducts (see Table 5 for details).

**Table 5:** Phosphorus recycling technologies and their second-generation P recovery rates

Technology	Feedstock	Pi and Po average recovery rate (%)
Extraction—Hedley fractionation	Animal manure	> 96
Struvite precipitation and crystallization technologies	Swine waste and wastewater	96.3
Hydrothermal treatments in the presence of acids	Swine manure	94
Aluminum-based water treatment residuals	Soil leachate, dairy wastewater, swine effluents	80–99
Thermochemical treatment	Sewage sludge	> 95
Nanofiltration and ion exchange method	Sewage sludge	90
Wet chemical extraction method	Sewage sludge ash	> 95
Hydrothermal treatment	Dewatered sewage sludge	98.37
Bacterial treatment	Sewage sludge ash	57
Electrochemical processes (e.g., electro-sorption, electro-coagulation, electro-induced precipitation, electrochemical crystallization)	Wastewater, sewage sludge, and sewage sludge ash	~ 90
Kiln	Bird feathers and bones from fish, swine, cattle, chicken, sheep	4–17
Composting or biological processes	Municipal mixed waste	94.2

*Pi;* inorganic P, *Po;* organic P, *%;* percentage

Composting organic waste, such as food scraps, manure, and crop residues, is an effective method for recycling P. During composting, microbial activity breaks down organic matter, releasing nutrients, including P, back into the soil. Vermicomposting, which uses earthworms to decompose organic waste, can further enhance P availability. These methods improve soil fertility and structure while reducing waste sent to landfills (Adhikari et al., 2020). Struvite (magnesium ammonium phosphate) precipitation is a well-established technique for recovering P from wastewater. In this process, magnesium, ammonia, and phosphate ions in wastewater react to form struvite crystals, which can be easily harvested and used as a slow-release fertilizer. Struvite precipitation is effective in wastewater treatment plants, reducing effluent P loads and recovering valuable nutrients (Ali et al., 2021). Acidic and alkaline leaching processes can extract P from solid waste materials, such as sewage sludge ash. These processes involve treating waste with acid or alkali solutions to dissolve P, which can then be recovered through precipitation or crystallization. While effective, these methods require careful chemical inputs and byproducts management to minimize environmental impacts (Yin et al., 2021). Thermochemical treatment involves heating waste materials, such as sewage sludge or animal manure, at high temperatures to recover P. This process can also destroy organic contaminants and pathogens, producing biochar rich in P and other nutrients. Thermochemical treatment efficiently recycles P from organic waste and improves soil health (Staroń et al., 2017). Nanofiltration and other membrane technologies can separate P from wastewater by selectively allowing water molecules to pass while retaining larger ions and molecules, including phosphate. These technologies are increasingly used in wastewater treatment plants for P recovery and water purification. They offer high efficiency and scalability, though initial investment and operational costs can be significant (Loganathan et al., 2017). Electrocoagulation involves applying an electric current to wastewater to destabilize and aggregate suspended particles, including P compounds, which can then be removed as sludge. This technique effectively treats industrial and municipal wastewater, offering a versatile and relatively low-cost option for P recovery (Bazrafshan et al., 2012). Electrochemical precipitation uses electrodes to induce the formation of P-containing precipitates, such as struvite, from wastewater. This method can be integrated with other treatment processes, providing a flexible approach to P recovery. Electrochemical techniques are gaining popularity due to their effectiveness in removing and recovering nutrients from complex wastewater matrices (Mayer et al., 2020).

Human waste represents a significant and underutilized source of recoverable P, which can be harnessed through various treatment and recovery technologies. Studies indicate that P excretion rates from human diets can lead to substantial urban P production, estimated at around 3.6 million tons annually (Kok et al., 2018; Cupisti and Gallieni 2018; McClure et al., 2019). This highlights the importance of wastewater as a resource, particularly as the demand for P continues to rise while natural reserves dwindle (Fattah et al., 2022). Advanced recovery methods, such as struvite precipitation, have demonstrated recovery rates exceeding 90% from wastewater, showcasing the feasibility of extracting P from this source (Fattah et al., 2022; Zhang et al., 2014). Moreover, integrating nutrient recovery technologies in wastewater treatment addresses P scarcity and mitigates environmental issues such as eutrophication caused by untreated waste (Egle et al., 2016). The recovery of P from human waste can play a critical role in creating a circular economy for nutrients, transforming waste into a valuable resource for agricultural use (Wirth et al., 2021; Yuan et al., 2012). Municipal wastewater treatment plants process human excreta, which contains significant quantities of P. Recycling P from municipal solid waste incineration could be an alternative to rock phosphate in fertilizer production (Kalmykova & Karlfeldt Fedje, 2013). However, safety and health regulations must be considered when handling and applying such waste-derived fertilizers to ensure food safety. The meat industry produces substantial waste, including feathers, bones, and poultry litter, which can be efficiently repurposed into valuable products. Feathers, rich in keratin, are processed into feather meal, a high-protein feed supplement for livestock. Bones can be rendered into bone meal, a key ingredient in animal feed and organic fertilizers, providing essential nutrients like calcium and P. Poultry litter is a mixture of manure and bedding. It is a nutrient-rich organic fertilizer source, promoting sustainable agriculture by enhancing soil fertility and reducing the need for chemical inputs. These innovative waste management practices contribute to circular economy principles and environmental sustainability. These materials are rich in nutrients, making them suitable for recycling into P-rich fertilizers. For example, thermal treatment of feathers and meat bone meal in a rotary kiln can produce ash with high P (4–17%), calcium (17–30%), and potassium (0.6–3.6%) content (Staroń et al., 2017). The thermal treatment of agricultural waste products can yield significant amounts of P and other essential nutrients, contributing to sustainable agriculture practices in the region (Staroń et al., 2017). Broiler and hog feed often consists of plant grains supplemented with feed phosphates. However, P in plant feed is primarily phytate bound, making it indigestible to animals. This results in excessive P in feed and inadequate absorption, leading to runoff and environmental contamination (Zwetsloot et al., 2015a, b; Staroń et al., 2017). To improve P absorption in livestock, farmers can explore various strategies. Plant breeders are developing grains with higher concentrations of bioavailable P and lower phytate-bound P. Additionally, adding phytase—an enzyme that breaks down phytate—to animal feed can enhance P digestibility. Balancing other nutrients in feed is also essential to optimize P availability (Mozhiarasi & Natarajan, 2022).

Phosphorus, which is crucial for plant development and achieving high crop yields, is increasingly obtained from fertilizer components, but due to fixation and microbial activity, only 20–30% of applied phosphate is utilized by crops (López-Arredondo et al., 2014). The remainder is lost in the soil, eventually contributing to runoff and eutrophication of waterways. Phosphorus runoff into waterways is a global issue, particularly in regions with intensive animal agriculture, such as the Mississippi River Basin in the USA (Stackpoole et al., 2021), the Netherlands'Rhine-Meuse-Scheldt Delta (Savenko & Savenko, 2022), and the Yangtze River Basin in China (Ji et al., 2024). These areas are hotspots for nutrient runoff due to concentrated livestock operations and high rates of fertilizer use, which contribute to water quality degradation, including eutrophication and harmful algal blooms in connected water bodies like the Gulf of Mexico, the North Sea, and the East China Sea. Addressing runoff in these regions requires coordinated agricultural and environmental management practices (Kleinman et al., 2015; Steinman et al., 2018). In 2017, a report highlighted unsafe *Escherichia coli* levels in the Shenandoah River (Virginia, USA), directly linked to manure runoff from surrounding livestock farms (Schaeffer et al., 2017). Other examples of rivers where P runoff has been particularly challenging include the Mississippi River (USA), Chesapeake Bay (USA), Yangtze River (China), Rhine River (Europe), Murray-Darling Basin (Australia), Ganges River (India), Congo River Basin (spanning six central and eastern African countries), and Amazon River Basin (Brazil) (Olde Venterink et al., 2003; Li et al., 2016; Bauters et al., 2021; Cunha et al., 2022; Sabo et al., 2022; Zhang et al., 2023; Varma and Jha 2024; Guo et al., 2025).

Excessive P application leads to eutrophication, causing harmful algal blooms and negatively impacting aquatic ecosystems and human health. Strict manure and fertilizer runoff regulations are necessary to mitigate these environmental impacts. In addition, wastewater disposal methods and treatment processes, such as settling, clarifying, and reducing biological and pathogen content, only have a limited impact on problem nutrients like P and nitrogen. Therefore, many communities now employ tertiary (advanced) treatment to reduce nutrient levels before discharge, including enhanced biological P removal, biofilm reactors, microalgae, and fungi-based systems, constructed wetlands, membrane bioreactors, chemical precipitation, advanced oxidation processes, biosorption, and struvite crystallization (Abdoli et al., 2024). Livestock farms contribute significantly to local and national economies, with North Carolina and Georgia leading in poultry production (USDA Allmon, 2016; NASS, 2017). Improving P digestion in livestock is crucial to reducing runoff and eutrophication. For instance, cow manure effectively enhances soil P and organic matter levels, whether natural or enhanced with limestone, gypsum, or additional P (Almeida et al., 2019). Arbuscular mycorrhizal fungi (AMF), phosphorus-mineralizing bacteria (PMB), and phosphorus-solubilizing bacteria (PSB) are being studied for their potential to fix phosphate in the soil, making it available to plants. This symbiotic relationship is akin to nitrogen fixation in legumes and can be harnessed to enhance P uptake and improve crop yields. For example, rice (*Oryza sativa*) can utilize AMF, PSB, or PMB to fix P in the soil, as can tarragon (*Artemisia dracunculus*), which utilizes AMF (López-Arredondo et al., 2014). These microorganisms can convert insoluble forms of P in soil into soluble forms that plants can absorb. Studies have shown that incorporating PSMs into agricultural practices can significantly enhance soil P availability and reduce the need for chemical fertilizers (García-Berumen et al., 2024; Richardson & Simpson, 2011).

Sustainable P recycling techniques offer promising alternatives to conventional PR mining, addressing the dual challenges of resource depletion and environmental degradation (Li et al., 2022). Recovering P from waste streams contributes to a circular economy and can enhance soil fertility and reduce pollution. Integrating biological, chemical, technological, and electrochemical recycling approaches can help achieve more sustainable P management, supporting agricultural productivity and environmental sustainability. Further research and development are needed to optimize these techniques and overcome practical challenges in implementation, including cost, scalability, and regulatory compliance.

## Phosphorus fixation: bioavailability and acquisition of phosphorus within the rhizosphere

### Forms of acquisition

Phosphate rock is not the sole source of P available for plant uptake because soil-bound P can also be converted into bioavailable forms through P fixation, making it readily accessible to plants. Phosphorus fixation is the process by which soil-bound P is converted into bioavailable forms, enabling plants to absorb it efficiently. Phosphorus can be fixed by either soil sorption or precipitation by free aluminum, iron, and calcium, as P can bind to these elements depending on the soil pH. Phosphorus in non-bioavailable forms can be fixed by PSB, PMB, as well as AMF to enhance P acquisition by plant root systems. For example, Konečný et al. (2019) explored the mechanism of P uptake from the soil through arbuscular mycorrhizal symbiosis and the genes and phosphate transporters essential for this process in AMF. They found that plants produce carbon and sugars via photosynthesis, and AMF can derive about 2–20% of their carbon from plants through a mutualistic relationship between AMF and plant roots. In this relationship, the fungi solubilize P and make it available to plants, facilitating a reciprocal exchange of nutrients. Therefore, soil P can be transformed from a non-bioavailable form into a form that plants can take up through phosphosolubilizing and phosphomineralizing bacteria and other microorganisms like AMF. For phosphate to become available to plants, hydrogen ions must be present to bond with the phosphate. Plants that release hydrogen ions in soils rich in phosphate (PO_4_^3^⁻) can facilitate the formation of bioavailable forms of P, such as monohydrogen phosphate ions (HPO_4_^2^⁻) and dihydrogen phosphate ions (H_2_PO_4_^⁻^). These processes involve P transporters and the formation of an AMF P-based symbiosis signaling pathway within the rhizosphere.

Studies such as those by Barrow (2022) provided an extensive overview of soil P dynamics, emphasizing the importance of considering both organic and inorganic P forms and their interactions with soil microorganisms. Barrow and Lambers (2022) highlight the role of microorganisms, such as PSB and PMB, in enhancing P solubilization and plant uptake, underscoring the symbiotic relationships that contribute to efficient P utilization in ecosystems. Gerke (2015) and Liu et al., (2022a, b) have extensively reviewed the most critical organic P compounds in soils, detailing how these compounds contribute to the overall P cycle and plant availability. The insights from these reviews are crucial for understanding the complexities of P bioavailability and acquisition.

The study by Luginbuehl and Oldroyd (2017) also reported the following phosphate transporters: phosphate transporter 1 (Pht1), PT4 (MtPT4), and PT13. They complement yeast phosphate transport mutants, branch the domain of the periarbuscular membrane, and function to sense phosphate levels, respectively. Other AM-specific phosphate transporters include MtPT1 - 3, MtPT4, MtPT5 - 6, and MtPT1-MtPT6 for *Medicago truncatula*; LjPT3 for *Lotus japonicus*; OsPT11/13 for *Oryza sativa*; PhPT4 for *Petunia* × *atkinsiana* (Sweet); NtPT5 for *Nicotiana tabacum*; StPT3/4/5 for *Solanum tuberosum*; LePT4/5 for *Solanum lycopersicum*; and HvPT8 for *Hordeum vulgare*.

### Mechanisms of phosphorus acquisition

#### Arbuscular mycorrhizal fungi (AMF)

**Fig. 3:**
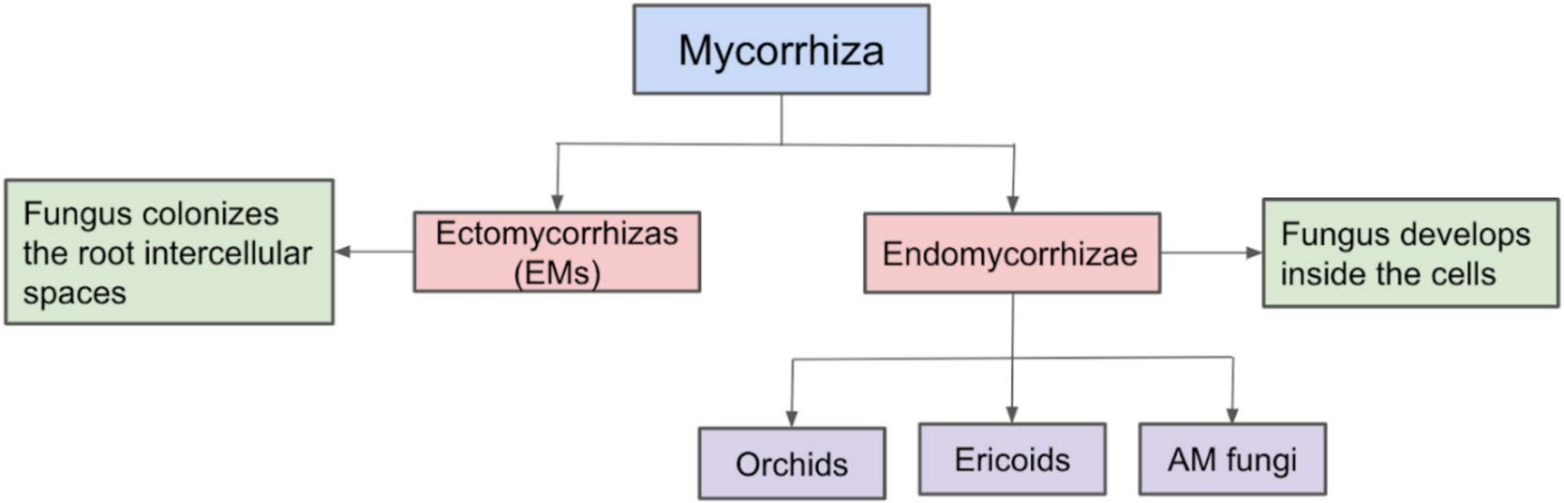
Classification of mycorrhizal fungi based on anatomy. Source: Adapted from Bonfante and Genre (2010)

There are different kinds of mycorrhizal fungi, and they can have different classifications according to their anatomy and where they develop concerning the dermal layer of the root and the specific individual root cells (Fig. 3). AM fungi penetrate roots via hyphae, forming arbuscules within root cells for nutrient exchange through specific transporters. Plant carbohydrates sustain mycorrhizal fungal colonization, and strigolactones aid in fungal branching and metabolism. The common symbiosis pathway facilitates root–fungi interactions, guiding arbuscule formation and nutrient exchange in the inner cortex. Arbuscules have short lifespans, leading to hyphal cell wall shrinkage and potential formation of new hyphopodia. The symbiotic interface allows safe fungal travel through plant cells, enhancing nutrient exchange without harming the host (Bonfante and Genre, 2008). Based on anatomy, the two main classifications for mycorrhizal fungi are ectomycorrhizae (EM), which colonize the intercellular region of roots, and endomycorrhizae, which develop within the plant cells. The latter can be classified into three subgroups: orchids, ericoids, and arbuscular mycorrhizae. Mycorrhizal fungi are essential for nutrient acquisition in plant root systems, facilitating nutrient and sugar exchange at symbiotic interfaces. Protein presence enables this process, with different fungal genomes displaying variations in gene numbers. Ectomycorrhizal (EM) and arbuscular mycorrhizal (AM) fungi have distinct colonization methods. EM fungi colonize the lateral portion of roots, forming a mantle and penetrating the dermal layer to exchange nutrients through the Hartig net.

It is also important to note that all AMFs are a monophyletic group belonging to the phylum Glomeromycota that can host endobacteria. It appears that both different kinds of fungi can transfer nutrients to and from plant roots, but their methods of colonization are different (Shi et al., 2022). EM fungi colonize the lateral portion of the root and coat it with a fungal mantle that encompasses the root tip. The EM fungi can penetrate through the outer dermal layer of the root, and then pass through the spaces between root cells, forming the Hartig net where nutrients can be exchanged. These EM fungi have a few enzymes that target components on the cell wall of the plant (e.g., pectin lyases and pectinases), and the genes that encode enzymes such as these can be upregulated as the EM fungi are developing inside of the plant’s tissue. On the other hand, AMF reproduces by spores and penetrates through the development of their hyphae, which can penetrate the dermal layer of the root in a concentrated area as opposed to generating a mantle that covers the root tip. The area where the hyphae penetrate the root is referred to as the hyphopodium and coats the small area of penetration outside of the root wall, and then the hyphae generate arbuscules within each cell in the root. Once the hyphae have contacted the cell wall of the root, it will begin to swell, flatten, and branch repeatedly to form the hyphopodium. This branching will occur near young lateral roots, and the hyphopodia will eventually protrude into the outer layer of the root’s epidermal layer. As the hyphopodium develops, the fungal growth will stop for about 4–6 h, and then the penetration hyphae will develop to continue growing inside epidermal cells to develop the arbuscules. It is on the surface area of the arbuscules where the exchange of nutrients occurs within the roots’ cells, and it is the specific Pi transporters that make this exchange of P and N nutrients from the AM fungi to the plant possible. This same area on the arbuscule allows the AMF to acquire carbohydrates from the plant, such as sucrose, glucose, and fructose, that can be processed through hexose transporters to form lipids and trehalose. The life cycle and colonization of mycorrhizal fungi are limited by the availability and uptake of carbon (sugars) that is provided by the host plant. Hence, the function of the hexose transporters is crucial for the continuation of mycorrhizal colonization.

AMF (e.g., *Glomus* sp. and *Rhizophagus* sp.) are obligate biotrophs and cannot produce carbon but rely on photosynthetically fixed carbon from plant roots. In turn, they increase bioavailable P acquisition by the plant by producing phosphatases and organic acids. AMF reproduce through spores, and once their hyphae begin to develop, they form symbiotic (nutrient exchange) relationships with plant roots by penetrating the cell walls of plant roots. Within these cells, the AMF will develop arbuscules, which are the location where the nutrient exchange occurs. The space between the cell wall and the arbuscule is known as the periarbuscular space. It often contains P transporters and hexose transporters that allow phosphate and sugars to move between both organisms, respectively. The AMF translocates the carbon to their external mycelia for their growth and development. In contrast, the plant xylem moves the P to the shoots and other parts where they are needed.

Carbohydrates play a crucial role in sustaining the colonization of roots by mycorrhizal fungi. Spores feed germinating hyphae through the breakdown of storage lipids for a short period until a suitable host is found. Strigolactones released by roots stimulate fungal branching and metabolism, aiding in establishing symbiotic relationships. Fungal exudates and calcium signaling facilitate hyphal penetration into the plant’s inner cortex, where arbuscules form for nutrient exchange. The SYM pathway contributes to successful symbiosis between the root, AMF, and nitrogen-fixing rhizobia, requiring at least seven SYM genes for symbiosis to occur. The symbiotic interface allows AMF to travel through plant cells without causing harm, facilitating nutrient exchange. AMF, particularly AMF, is key in P acquisition in symbiotic relationships with plant roots. However, factors such as fertilizer application can affect interactions between AMF communities and plants, potentially disrupting the symbiotic relationship and leading to a decline in AMF colonization.

The production of AMF contributes to the spread and increase of P availability and nutrition as well as the plant’s ability to depolymerize organic polymers from dead microbial biomass use to the ability of the plant to produce (Ganugi et al., 2019; Garcia-Garrido et al., 2000; Leigh et al., 2008). An example is reported by Li et al. (2006), where the mycorrhizal fungus *Rhizophagus irregularis* inoculation onto spring wheat (*Triticum aestivum*) supported a contribution of more than 50% of the P absorption by the plant. A similar relationship was also found with *Funneliformis mosseae* and durum wheat (*Triticum durum*, cv. *Petra*), and *Claroideoglomus etunicatum* inoculated plants (*Triticum aestivum*, cv. *Otto*) where significant differences in P acquisition and uptakes were recorded and a correlation in increased plant dependency on mycorrhizae and mineralizing phosphatase (P-ase) within the AM-fungus-colonized roots (Al-Karaki, 2002; Li et al., 2006). Synergistic interactions between bacteria (like *Streptomyces* sp.) and plant-growth-promoting rhizobacteria (*Azotobacter chroococcum* with *Bacillus* spp.) and AMF (*Rhizophagus fasciculatus*) for P uptake have also been proven experimentally (Battini et al., 2017; Ganugi et al., 2019; Khan & Zaidi, 2007) and reflect the beneficial mineralizing phosphatase (P-ase) effect of the AM-fungus-colonized roots. AMF contributes to phosphorous absorption, nutrient cycling, heavy metal and saline tolerance, and protection from root diseases (Augé, 2001; Feng et al., 2002; Govindarajulu et al., 2005; Lambert et al., 1979; Linderman, 2000). Regarding their classification, the complementary use of morphological and molecular methods will aid in understanding mycorrhiza-specific genes or gene families that aid their mechanistic roles within the rhizosphere (Lee et al., 2013; Liu & Feng, 2010). Arbuscular mycorrhizal fungi (AMF) are grouped mainly in the monophyletic phylum Glomeromycota and are primarily obligate biotrophs (Stürmer, 2012).

#### Phosphate-solubilizing bacteria (PSB) and phosphate-mineralizing bacteria (PMB)

Phosphobacteria enhances the availability of P in the soil for plant uptake, serving as biofertilizers through their P-mineralizing enzymes and the bacterial genes that encode for enzymes capable of promoting phosphate acquisition in plants (Castagno et al., 2021). PSBs (e.g., *Enterobacter* sp. and *Pseudomonas* sp.) and PMBs (e.g., *Burkholderia* sp. and *Bacillus* sp.) are considered plant-growth-promoting bacteria because they enhance plant P acquisition. Some other soil bacteria that have been reported to mobilize poorly available P via solubilization and mineralization include *Pseudomonas* spp., *Agrobacterium* spp., and *Bacillus circulans* (Babalola & Glick, 2012). Table 6 outlines various phosphobacteria species, their types, associated plant species, and growth/yield parameters improved by these bacteria.

**Table 6:** Phosphobacteria species and their yield parameters

Phosphobacteria	Bacterial type	Plant	Growth/yield parameters improved by phosphobacteria	References
*Enterobacter* sp. MS32, *Pseudomonas* sp. MS16	PSB	*Triticum aestivum*	Increased grain yield (38.5% and 17–18%, respectively)	Suleman et al. (2018)
*Pantoea cypripedii* (PSB- 3), *Pseudomonas plecoglossicida* (PSB- 5)	PSB	*Triticum aestivum* and* Zea mays*	Increased yield in maize (20%) and in wheat (16%)	Kaur and Reddy (2015)
*Pseudomonas fluorescens*, *Serratia* sp.	PSB	*Triticum aestivum*	Increased P uptake (> 50%)	Schoebitz et al. (2013)
*Pseudomonas tolaasii* IEXb	PSB	*Zea mays*	Enhanced seedling emergence (8%), shoot length (19%), grain yield (44%), 1000-grain weight (18%), plant biomass (32%), and P content	Viruel et al. (2014)
*Acinetobacter* L176, *Bacillus* sp. L55, *Enterococcus* sp. L191, *Pantoea* sp. J49, *Serratia* sp. S119	PSB	*Arachis hypogaea* and* Zea mays*	Increased plant aerial length (up to 2.1-fold) and an increase in the total aerial P and N content. Increased growth parameters and overall P content in soil and inoculated plants	Anzuay et al. (2015)
*Bacillus megaterium* YM13, *Ensifer adhaerens* TPMX5, *Enterobacter* sp. YM14, *Providencia rettgeri* TPM23	PSB	*Arachis hypogaea*	Increased root length (25–49%), stem length (19–28%), and number of leaves (12–37%). Growth-promoting effects enhanced by the addition of tri-calcium phosphate	Jian et al. (2019)
*Acinetobacter pittii* JD- 14, *Bacillus* sp., *Enterobacter* sp.	PSB	*Medicago sativa*	Improvement of different growth parameters and nutrient content, including P, fresh and dry weight, and increase (41 and 34%, respectively)	Daur et al. (2018)
*Pantoea eucalypti* M91	PSB	*Lotus tenuis*	Increased dry matter yield (68%) and P concentration (15.4%) at early growth stages	Castagno et al. (2021)
*Bradyhizobium japonicum* + *Pseudomonas putida*	PSB	*Glycine max*	Improved growth and nodulation	Rosas et al. (2006)
*Mesorhizobium mediterranean* PECA21	PSB	*Cicer arietinum* and *Hordeum vulgare*	Increased dry weight (56%), and nitrogen and P content (onefold) in barley. Increased dry weight (18%) and nitrogen and P content (1.25-fold) in chickpeas. Increased potassium, calcium, and magnesium and content in both species	Peix et al. (2001)
*Enterobacter aerogenes* R4M-A	PSB	*Phaseolus vulgaris*	Increased plant dry biomass (1.7-fold) and nitrogen content in leaves	Collavino et al. (2010)
*Pseudomonas* sp. CCAR59	PMB	*Triticum aestivum*	Increase phytate-derived P availability. Shoot dry-weight-like plants fertilized with soluble P	Richardson et al. (2000)
*Burkholderia* sp. FpRpG4	PMB	*Lotus japonicus*	Increased shoot dry weight (threefold) and length (sixfold)	Unno et al. (2005)
*Bacillus amyloliquefaciens* FZB45	PMB	*Zea mays*	Enhanced plant growth and chlorophyll content (sixfold)	Idriss et al. (2002)
*Bacillus* sp. Z- 014	PSB	*Triticum aestivum*	Enhanced plant growth and P availability	Liu et al., (2022a, b)
*Bacillus mucilaginous D4B1* (WT), NKTS- 3 (a transgenic strain that produces highly active phytase)	PMB	*Nicotiana tabacum*	Plant growth increased by both strains in pot (NKTS- 3, 2.3-fold; WT, 1.8-fold) and field trials (NKTS- 3, 1.2-fold; WT, 1.1-fold)	Li et al. (2007)

#### AMF, PSB, and PMB

Zhang et al. (2016) conducted a study on the interactions between arbuscular mycorrhizal fungi (AMF) and phosphate-solubilizing bacteria (PSB), exploring their synergistic roles in P acquisition. The study used two host plants, *Medicago sativa* and *Daucus carota* L., each paired with a specific AMF strain and the same PSB strain (Table 7). Although AMFs are essential for phosphorus uptake, they cannot secrete phosphatases needed to solubilize phosphate (Tisserant et al., 2013). According to Zhang et al. (2016), the presence of these exudates suggests that PSBs can utilize compounds released by AMF hyphae, enhancing P solubilization. PSBs benefit from AMF exudates and highlight a potential symbiotic relationship where PSBs provide inorganic P to AMFs, supporting plant growth. It is unknown if the release of carbon compounds from AMF hyphae is under AMF control or if PSBs benefit from carbon leakage. While Zhang et al. (2016) demonstrated the relationship between AMF, PSB, and host plants, the limited scope (using only RIn, RIr, and RA strains) suggests further research to understand these interactions comprehensively. Fungal mycelium networks significantly increase inorganic P acquisition compared to root systems alone. Fungi can recruit PSBs to make organic P available for plant uptake, with the interactions between AMF and PSB influenced by the P status in the environment (Bhalla et al., 2022).

**Table 7:** Plant host, AMF, and PSB relationships

Host plant	AMF	PSB	Reference
*Medicago sativa*	*Rhizophagus intraradices* BEG 141 (RIn)	*Rahnella aquatilis* HX2 (RA)	Zhang et al. (2016)
*Daucus carota* L. roots (transformed)	*Rhizophagus irregularis* DAOM 197198 (RIr)	*Rahnella aquatilis* HX2 (RA)	Zhang et al. (2016)

### Phosphorus regulations

The work of Barrow (2022) highlighted the challenges in making soil P bioavailable to plants. Phosphorus in soils often exists in insoluble forms that plants cannot readily absorb. The solubilization of P is a critical step that involves converting these insoluble forms into soluble ones that plant roots can take up. This process is facilitated by soil microorganisms, including phosphate-solubilizing bacteria (PSB) and arbuscular mycorrhizal fungi (AMF), which release organic acids and enzymes that dissolve phosphate compounds, thereby enhancing P availability to plants (Barrow & Lambers, 2022). Gerke (2015) suggested that organic P compounds, such as inositol phosphates (phytates), are significant soil P reservoirs. However, their availability to plants depends on the activity of phosphatases—enzymes that catalyze the hydrolysis of organic phosphates into inorganic forms, underscoring the importance of maintaining a healthy microbial community in the soil. Liu et al., (2022a, b) elaborated on the transformation processes of organic P compounds and their implications for soil fertility. They highlighted that organic P can be mineralized through microbial action, releasing inorganic phosphate ions that plants can assimilate. This mineralization process is influenced by various factors, including soil pH, moisture, and organic matter content, which affect microbial activity and enzyme production.

Bhalla et al. (2022) emphasized the critical role of phosphate sensing in plant immune systems, stress responses, and cell surface regulation, highlighting phosphate homeostasis as a vital process for facilitating communication and metabolism in fungi. Inorganic phosphate homeostasis influences interactions with bacteria, growth states, stress adaptations, and phosphate-starvation responses. Also, phytohormones and microRNAs, such as miR399, play significant roles in regulating phosphate-starvation responses, thereby enhancing phosphate acquisition from soils. Pantigoso et al. (2020) compared P levels in cultivated and non-cultivated potatoes and examined the microbial communities in their rhizospheres. They used a crop domestication gradient to assess P impact and found that differences in P levels were associated with the presence or absence of phosphatase and specific genotypes influencing rhizosphere microbial communities. The study suggested that excessive P application in soils for cultivated plants could disrupt interactions between plants and microbes necessary for converting P to its bioavailable form. Inorganic phosphate can inhibit microbial phosphatase enzyme synthesis and activity, which is essential for P mineralization and leads to P immobilization through adsorption or conversion into organic forms like phytic acid.

Mutucumarana et al. (2014) evaluated nonphytate P, digestible P, and retainable P contents in broiler chicken feeds with varying nonphytate P levels. Their findings revealed higher P concentrations in corn-based diets than calculated values and varying calcium concentrations. Increasing dietary P concentrations linearly increased ileal and excreta P outputs, showing distinct patterns in corn- and canola meal–based diets. Strong correlations between dietary P intake and digest/excreta P outputs allowed the determination of true P digestibility coefficients for both diets. Lee et al. (2023) compared plant-based and animal-origin P sources in animal feed, addressing the challenges of digesting phytate-bound P in plant grains. The study examined feed phosphates from PR, analyzed P and calcium concentrations in dicalcium and monocalcium phosphate, and emphasized the importance of considering P digestibility in animal feed formulations to optimize nutrient utilization. Konečný et al. (2019) studied P uptake by arbuscular mycorrhizal symbiosis, focusing on the genes and phosphate transporters necessary for P uptake by AMF, using *Medicago truncatula* as the primary plant model. Plants provide carbon and sugars through photosynthesis, whereas AMF, lacking photosynthetic capability, can only obtain about 2–20% of their carbon from plants. This mutualistic relationship allows plants to supply carbon to fungi via roots while fungi solubilize P, making it available to plants.

Arbuscules play a crucial role in regulating P uptake in plants, with symbiotic phosphate transporter proteins facilitating phosphate transfer to plant cells for nutrient exchange. The nutrient exchange process involves colonization mechanisms and induction of phosphate transporter genes by the host plant to acquire P from arbuscules. While various proteins are required, the symbiotic status of mycorrhizal fungi is not limited by their number of protein-coding genes. Notably, *T. melanosporum*, with a 125-mb genome containing 7500 protein-encoding genes, was found to have a larger genome than *Laccaria bicolor*, which had a 65-Mb genome with approximately 19,102 protein-encoding genes (Martin et al., 2008). The expansion of specific gene families in *L. bicolor* allowed for more protein–protein interactions, facilitating signal-transduction mechanisms. A protein expressed on the periarbuscular membrane enables nutrient and sugar exchange at the symbiotic interface between mycorrhizal fungi and plant roots, functioning as a control system for nutrient transfer (Harrison et al., 2002). The mycorrhizal and direct pathways play roles in nutrient acquisition and exchange, though the mechanisms that balance these pathways remain unclear (Smith et al., 2011). Host plant genetic composition can significantly affect AMF communities hosted (Kiers et al., 2011).

Kobae (2019) noted that tillage might disturb AMF communities but also promote regeneration by selecting rapidly colonizing fungi that produce numerous spores. Spore-based RNA sequencing differentiated AMF taxa, classifying them into genera such as *Paraglomus*, *Ambispora*, and *Diversispora*. Some AMFs produce vesicles in roots as arbuscules senesce, with numbers increasing in dying and aging roots, although their specific role is not fully understood. These vesicles contain nuclei, glycogen granules, small vacuoles, and lipid droplets in their protoplasms. The formation of the periarbuscular membrane requires the t-SNARE protein SYP132 A. Temporal expression of transporter proteins impacts arbuscular growth, while arbuscule shrinkage or degradation regulates processes protecting host plant cells from damage. Extraradical fungal mycelium mineralizes soil nutrients, and high-affinity phosphate and ammonium transporters facilitate nutrient uptake. Phosphate and nitrogen transfer to arbuscules occurs as polyphosphate and arginine, released into the periarbuscular space as phosphate and ammonium. Kobae (2019) raised questions about the significance of fungal transporters in releasing nutrients into the periarbuscular space.

Plant phosphate transporters like *Pht1* can complement yeast phosphate transport mutants, while MtPT4 transporters in *M. truncatula* are involved in the branching domain of periarbuscular membranes, crucial for transferring phosphate from fungi to plants. Another transporter, *PT13*, induced by mycorrhizal association in rice, may not transport phosphate directly but plays a role in sensing phosphate levels. Nutrient transport involves creating an electrochemical gradient through proton transport. Kobae (2019) highlighted fungi’s preference for glucose over sucrose, emphasizing apoplastic invertases and sucrose synthase’s importance in mycorrhized roots, where cleaved sucrose serves as fungi’s favored carbohydrate source. Lipids are crucial for fungal structure formation in nutrient uptake and essential for hyphae germination and colonization. Understanding molecular processes in fungal root colonization can benefit from tools like host-induced gene silencing and access to genome and transcriptome sequences.

## Conclusion

Phosphorus is a crucial nutrient for plant growth and agricultural productivity, yet its management poses significant environmental challenges. The mining of PR, a non-renewable resource, is the primary means of obtaining P for agriculture, but it brings about habitat destruction, water pollution, and substantial energy consumption. Moreover, the finite nature of PR reserves raises concerns about the long-term sustainability of P supply for global agriculture. The use of mined P in agriculture often leads to low plant uptake efficiency, with a substantial portion of applied P immobilized in the soil. This immobilization contributes to P runoff into water bodies, causing eutrophication, algal blooms, and subsequent water quality degradation. Therefore, effective P management is imperative to minimize these environmental impacts and ensure agricultural sustainability. To address challenges associated with low plant uptake efficiency, P immobilization in soil, environmental impacts like eutrophication and algal blooms, water quality degradation, reliance on finite mined P resources, uneven global distribution of reserves, increased economic costs for farmers, and sustainability concerns, several sustainable P management strategies can be adopted. The strategy includes precision farming, nutrient management planning, development of P-efficient crop varieties, recycling P from organic waste streams (e.g., manure and crop residues), enhancing P bioavailability through microbial processes such as mineralization and mycorrhizal symbiosis, optimizing fertilizer application methods, and promoting struvite recovery from wastewater for use as a sustainable P source. Recycling P from organic waste streams, such as animal manure and crop residues, can reduce dependence on mined P and mitigate environmental impacts. The use of microbial processes, including microbial mineralization and mycorrhizal symbiosis, can enhance the bioavailability of P in soils, improving plant uptake efficiency and reducing the need for synthetic fertilizers.

Recent research has emphasized the role of microorganisms in P solubilization and plant uptake. These microorganisms, including PSBs, PMBs, and AMFs, play a vital role in converting insoluble P forms into bioavailable forms, enhancing P acquisition by plants. In addition to microbial strategies, the development of biofertilizers can significantly enhance P availability in soils, promoting sustainable agricultural practices. By incorporating these strategies, agricultural systems can reduce their environmental footprint and contribute to a more sustainable future. The legacy contaminants associated with mined P, including ionizing radiation and heavy metals, present additional challenges to human and environmental health. These issues, coupled with the land fragmentation and degradation resulting from mining activities, underscore the need for stringent regulations and restrictions on P mining. Recycling P from organic waste materials is more cost-effective and environmentally sustainable than continuing mining PR. To mitigate the ecological impacts and evolutionary legacies of P mining, environmental impact assessments and reclamation efforts should be prioritized. Future research should focus on understanding the rhizospheric processes and mechanisms involved in P acquisition by crops, particularly through exploring intra- and interkingdom communication vehicles like extracellular vesicles. This review has contributed toward ensuring sustainable and resilient food and environmental systems and enhancing human health security. Additionally, the formulation and use of phosphobacterium biofertilizers, leveraging AMFs, PMBs, and PSBs, should be considered to improve P management in agriculture.

## Data Availability

No datasets were generated or analysed during the current study.
